# The flexibility of SABRE, a new quantitative receptor function model, when fitting challenging concentration-effect data

**DOI:** 10.3389/fphar.2025.1591761

**Published:** 2025-06-12

**Authors:** Barbara Olah, Vera Tarjanyi, Barbara Takacs, Edua Pluzsnyik, Gabor Viczjan, Ignac Ovari, Zoltan Szilvassy, Bela Juhasz, Judit Zsuga, Tamas Erdei, Rudolf Gesztelyi

**Affiliations:** ^1^ Department of Orthodontics, Faculty of Dentistry, University of Debrecen, Debrecen, Hungary; ^2^ University of Debrecen, Doctoral School of Nutrition and Food Sciences, Debrecen, Hungary; ^3^ Department of Pharmacology and Pharmacotherapy, Faculty of Medicine, University of Debrecen, Debrecen, Hungary; ^4^ Department of Psychiatry, Faculty of Medicine, University of Debrecen, Debrecen, Hungary

**Keywords:** SABRE model, operational model, furchgott method, curve fitting, atrium, inotropy

## Abstract

The Signal Amplification, Binding affinity, and Receptor-activation Efficacy (SABRE) model is the most recent general and quantitative model of receptor function, which enables the determination of K_d_ (the equilibrium dissociation constant of the agonist-receptor complex) and q (the fraction of the operable receptors after a partial irreversible receptor inactivation) from purely functional data. The practical aim of the present study was to test the capabilities of this new model using concentration-effect (E/c) data from a previous investigation conducted in our laboratory. We have found that the SABRE model is at least as useful as two widely accepted older methods thought to have similar capabilities, the operational model of agonism and Furchgott’s method, even if the quality of the data to be evaluated is somewhat challenging. Nevertheless, the SABRE model seems to require a large amount of high-quality and, regarding the experimental design, diverse data. In addition, it is important to find the most suitable fitting strategy for the particular sort of data in order to obtain reliable results. However, owing to its unique feature of distinguishing between receptor activation and activation of postreceptorial signaling, the SABRE model appears to be superior to previous quantitative receptor function models in simulating E/c curves and thereby clarifying, explaining or simply illustrating theoretical issues.

## 1 Introduction

The Hill equation is the first general and quantitative model of the function of receptors and their tissue-dependent postreceptorial signaling machinery (Weiss, 1997; Goutelle et al., 2008; Gesztelyi et al., 2012a; Finlay et al., 2020). The Hill equation was originally developed to describe the simplest manifestation of the ligand-receptor interaction, i.e., the ligand-receptor complex formation (Hill, 1910). The officially recommended name of this latter relationship is “Hill-Langmuir equation” (Neubig et al., 2003). The K_d_ (or K) parameter of the Hill-Langmuir equation, which is borrowed from chemical reaction kinetics, has an exact physico-chemical meaning, i.e., the equilibrium dissociation constant of the ligand-receptor complex (see: the Equation 1 below). If the Hill equation is used to describe a ligand-receptor interaction that results in a biological change, it is worth renaming K_d_ to EC_50_ (or ED_50_), because this parameter tends to lose its original meaning and to become an empirical constant. Accordingly, the EC_50_ (or ED_50_) expresses the ligand concentration (or dose) that elicits half of the maximal effect that is elicitable by the given ligand (which ligand is an agonist in this case) (Giraldo et al., 2002; Gesztelyi et al., 2012a). A common ambition of developers of new receptor function models has been being to keep K_d_ (with its exact physico-chemical meaning) and to condense the further contribution of the biological system (the receptor and the receptor-holding cell/tissue) into one (or more) additional parameter(s).

It is now widely accepted that, from the formation of the agonist-receptor complex to the induced biological change, at least two main events should be distinguished: 1) conformational change of the receptor (i.e., receptor activation), and 2) conformational and/or chemical alterations of further molecules in the cell/tissue (viz. activation of the postreceptorial signaling). However, the general and quantitative receptor function models (“receptor theory models”) possessed only one parameter to consider the contribution of the biological system (Ruffolo, 1982; Colquhoun, 1998; Giraldo et al., 2002; Shang et al., 2019; Kenakin, 2022), until the emergence of the Signal Amplification, Binding affinity, and Receptor-activation Efficacy (SABRE) model (Buchwald, 2017; Buchwald, 2019). The SABRE model is the most recent general and quantitative model of the function of the ligand-receptor-cell/tissue unit (Buchwald, 2017; Buchwald, 2019; Buchwald, 2020).

To understand its essence, it is worth surveying the evolution of the SABRE model up to a moderately complex form (used in the present study as well). If an agonist is able to fully activate its receptor (thus, it is a full agonist) and there is no postreceptorial signal amplification (or attenuation) in the system, then even the formula used for the Hill-Langmuir equation is suitable for describing the response of the biological system (Gesztelyi et al., 2012a). This relationship can be expressed with E/E_max_ as the dependent variable, which allows the number of fitted parameters to be reduced (Buchwald, 2017; Buchwald, 2019; Buchwald, 2020; Buchwald, 2022; Buchwald, 2023; Buchwald, 2025):

$$\frac{E}{E_{\max}}=\frac{c^{n}}{c^{n}+{K_{d}}^{n}}$$ (1)

where: c: the concentration of an agonist that serves as the independent variable (x value); E/E_max_: the fractional effect that is here the dependent variable (y value), computed as the ratio of E, the effect evoked by c, to E_max_, the maximal effect achievable in the given biological system (in practice, the maximal effect elicited by the strongest agonist in a series of experiments); K_d_: the equilibrium dissociation constant of the agonist-receptor complex that characterizes the binding affinity (in practice, this is an approximation of the chemical measure in question); n: the Hill coefficient (slope factor).

It cannot be overemphasized that, in the SABRE model as well as throughout this work, E_max_ is defined as the maximal effect achievable in the system and not that reachable by the agonist used. Due to this definition, and in order to preserve the parameter K_d_, the Equation 1 must be extended if we want to treat partial agonism and postreceptorial signal handling (attenuation or amplification) as well. It is also important to highlight that the SABRE model divides the signal handling of a biological system into a receptorial and a postreceptorial component and treats them separately (see below). Within the framework of the SABRE model, the terms signal attenuation and signal amplification are applied exclusively to the postreceptorial component of signal handling (Buchwald, 2017; Buchwald, 2019; Buchwald, 2020; Buchwald, 2022; Buchwald, 2023; Buchwald, 2025). For clarity, this will also be emphasized throughout this paper.

If an agonist (even at high concentrations) can only induce an incomplete receptor activation (so, it is a partial agonist) in a biological system with neutral postreceptorial signal handling (neither attenuation nor amplification), it cannot reach E_max_. To address partial agonism (so, to account for the receptorial handling of the signal), the SABRE model introduces an ε parameter (Buchwald, 2017; Buchwald, 2020), providing an equation similar to that of Ariëns (Ariëns, 1954; Ruffolo, 1982):

$$\frac{E}{E_{\max}}=\varepsilon \cdot \frac{c^{n}}{c^{n}+{K_{d}}^{n}}$$ (2)

where the new parameters: ε: the “receptor-activation” efficacy of the agonist (0 ≤ ε ≤ 1) that characterizes the ability of the agonist (once bound) to switch the receptor from an inactive conformation into an active one; n: a Hill-type coefficient (very similar to the classical one).

In fact, ε also characterizes the activity of the given active state, which becomes apparent when more than one active state exist. In this latter case, ε, determined this way, expresses the relevant properties of the agonist and the active states (including the relative time spent in these states) as a single resultant value. Otherwise, this receptor-activation efficacy (ε) is reminiscent not only of Ariëns’ intrinsic activity (α, defined to range from 0 to 1), but also of Stephenson’s drug efficacy (ε) and Furchgott’s intrinsic efficacy (ε_0_; both latter ones–ε and ε_0_ – are defined from 0 to ∞ ) (Ruffolo, 1982).

To account for the postreceptorial signal handling, the SABRE model introduces another parameter, γ. If the agonist can fully activate the receptor (i.e., when ε, the receptor-activation efficacy, is constant and maximal), the model takes the following form (Buchwald, 2017; 2020):

$$\frac{E}{E_{\max}}=\frac{{\gamma \cdot c}^{n}}{{\gamma \cdot c}^{n}+{K_{d}}^{n}}=\frac{c^{n}}{c^{n}+\frac{{K_{d}}^{n}}{\gamma }}$$ (3)

where the new parameter: γ: the gain factor of the postreceptorial signaling to describe attenuation (0 ≤ γ < 1) or amplification (γ > 1) or lack thereof (γ = 1).

Of course, similarly to ε, γ is also an average measure. It characterizes the efficiency of the processes induced by the active form(s) of the receptor regarding the biological response measured. The Equation 3 (especially the expression on the right-hand side of the equation) slightly resembles the forms of the Hill equation, which are modified with equations of Gaddum or Schild to account for the presence of an antagonist (Waud et al., 1978; Motulsky and Christopoulos, 2004; Gesztelyi et al., 2012b).

In turn, to allow partial agonism and postreceptorial signal handling simultaneously, the SABRE model provides a complex formula that differs considerably from the previous general and quantitative receptor function models (Buchwald, 2017; Buchwald, 2020):

$$\frac{E}{E_{\max}}=\frac{\varepsilon \cdot {\gamma \cdot c}^{n}}{{\left(\varepsilon \cdot \gamma -\varepsilon +1\right)\cdot c}^{n}+{K_{d}}^{n}}$$ (4)

Thus, the SABRE model assumes a complex relationship between the activation of the receptor and the activation of the postreceptorial signaling (in terms of their effects on the response of a biological system). This is reflected by the fact that γ appears exclusively as ε∙γ (and never alone), while ε occurs only once alone and twice as ε∙γ. Nevertheless, the SABRE model is the only general and quantitative model of receptor function that distinguishes between the contribution of the receptor and that of the postreceptorial signaling to the response of the whole system. In the SABRE model, the receptor activation is attributed to the ligand-receptor interaction, while the term signal amplification (or attenuation) refers to the operation of the postreceptorial signaling. The Equation 4 is a typical form of the SABRE model, although it is not the most general one (Buchwald, 2017; Buchwald, 2019; Buchwald, 2020; Buchwald, 2022; Buchwald, 2023). Nevertheless, for our current purposes, the Equation 4 appeared to be suitable and sufficient.

Numerous analytical methods have been established to determine K_d_ values, including radioligand binding assay (RBA), surface plasmon resonance, fluorescence resonance energy transfer method (FRET), affinity chromatography, affinity electrophoresis (e.g., electrophoretic mobility shift assay), protein-induced fluorescence enhancement and isothermal titration calorimetry (Ma et al., 2018; Lee et al., 2024). However, only few methods are available to assess K_d_ in living systems (*in vivo* or *ex vivo*), such as RBA (for cells and tissue slices), FRET (for cells) and the so-called functional assays (which utilize measurement data of a biological function obtained from any hierarchical level of a living organism). The SABRE model (by means of Equations 4, 5, see in the next section) has been reported to be suitable for determining K_d_ from purely functional data, specifically from concentration-effect (E/c) curves constructed in the absence and presence of a pretreatment with an irreversible antagonist (Buchwald, 2019; Buchwald, 2022).

It is generally accepted that comparative functional investigations carried out on tissues with naïve and depleted receptor populations are only reliable if the maximal response (evocable by the given agonist) assessed in the receptor-depleted tissue is significantly smaller than that measured in the naïve tissue (Motulsky and Christopoulos, 2004). However, in an earlier study, we found that the irreversible A_1_ adenosine receptor antagonist FSCPX (8-cyclopentyl-*N* ^*3*^-[3-(4-(fluorosulfonyl)benzoyloxy)propyl]-*N* ^*1*^-propylxanthine) was unable to significantly reduce the maximal direct negative inotropic response to three A_1_ adenosine receptor full agonists, namely, NECA (5′-(*N*-ethylcarboxamido)adenosine), CPA (*N* ^*6*^-cyclopentyladenosine) and CHA (*N* ^*6*^-cyclohexyladenosine), in the guinea pig atrium (Gesztelyi et al., 2013). In a later investigation, we have confirmed the inability of FSCPX to decrease the maximal direct negative inotropy mediated by the A_1_ adenosine receptor, with the use of different experimental protocols, in both guinea pig and rat atria (Viczjan et al., 2021). During the earlier study (Gesztelyi et al., 2013), K_d_ was determined for NECA, CPA and CHA using two standard functional assays, the operational model (Black and Leff, 1983) and Furchgott’s method (Furchgott, 1966; Furchgott and Bursztyn, 1967). Although these two procedures yielded congruent results, these estimates of K_d_ were considerably higher than those of others (provided by ligand binding assays and Furchgott’s method) (Gesztelyi et al., 2013). The goal of the present study was to explore how the SABRE model could cope with the challenging E/c data of our above-mentioned earlier study, in comparison with the two most important rival functional assays.

## 2 Materials and equipment

In the present study, we reevaluated the raw E/c data of a previous study from our laboratory (Gesztelyi et al., 2013). The E/c curves in question were constructed with NECA, CPA and CHA, generally thought to be A_1_ adenosine receptor full agonists (Fredholm et al., 2001; Elzein and Zablocki, 2008; Deb et al., 2019). As an output, the contractile force of isolated, paced guinea pig left atria was measured. The E/c curves were generated in the absence (labelled with “N” as naïve) and presence (labelled with “X”) of a pretreatment with FSCPX, an irreversible A_1_ adenosine receptor antagonist. The combination of the three agonists with the two pretreatment outcomes resulted in six groups (data sets): NECA N, NECA X, CPA N, CPA X, CHA N and CHA X (Gesztelyi et al., 2013).

For the present study, all effect values of the previous work (i.e., the percentage decrease of the initial contractile force: Gesztelyi et al., 2013) were divided by the biggest one of E_max_ values determined for the original data sets lacking FSCPX pretreatment *via* fitting the Hill equation (see: Supplementary Appendix). The biggest E_max_ value was 97.92 (%) that belonged to the NECA N group. Then, the fractional effect values obtained this way were multiplied by 100 to get percentage effect values. These recalculated E/c curves (in fact: E/E_max_ % vs. logc curves) were used here for all analyses.

## 3 Methods

### 3.1 Regression strategies with the SABRE model

**FIGURE 1:**
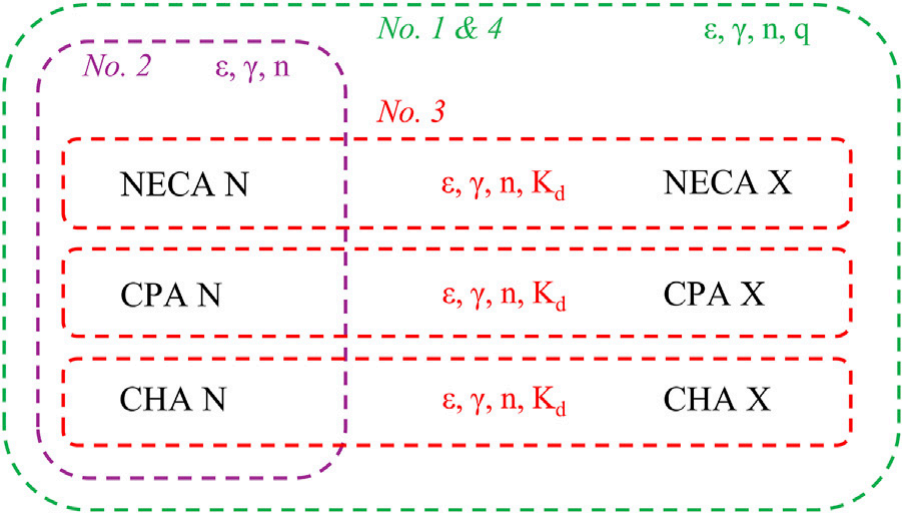
Strategies for fitting the SABRE receptor function model to six data sets, consisting of E/c curves of three synthetic A_1_ adenosine receptor full (or close to full) agonists (NECA, CPA, CHA), constructed in isolated, paced guinea pig left atria, in the absence (“N” labeling) or presence (“X” labeling) of a pretreatment with FSCPX, an irreversible A_1_ adenosine receptor antagonist (raw data obtained from Gesztelyi et al., 2013). The SABRE parameters, shared between (or among) the data sets, are marked in the same color as the dashed line encircling the concerned data sets. According to the rules of the multi-model global fitting, the parameter q, if shared, is shared only among the “X” labelled data sets (see: Supplementary Appendix). Green dotted line: first and fourth fitting strategies; Purple dotted line: second fitting strategy; Red dotted lines: third fitting strategy; E/c: concentration-effect; SABRE: Signal Amplification, Binding affinity, and Receptor-activation Efficacy (for an explanation of the SABRE parameters, see: Equations 4, 5); NECA: 5′-(*N*-ethylcarboxamido)adenosine; CPA: *N* ^*6*^-cyclopentyladenosine; CHA: *N* ^*6*^-cyclohexyladenosine; FSCPX: 8-cyclopentyl-*N* ^*3*^-[3-(4-(fluorosulfonyl)benzoyloxy)propyl]-*N* ^*1*^-propylxanthine.

The regression using the SABRE model was carried out following four fitting strategies (Figure 1).

For the first fitting strategy, all E/c curves were simultaneously fitted to two models, Equation 4 (see above) and Equation 5 (as follows; Buchwald, 2019; Buchwald, 2022):

$$\frac{E}{E_{\max}}=\frac{q\cdot \varepsilon \cdot {\gamma \cdot c}^{n}}{{\left(q\cdot \varepsilon \cdot \gamma -q\cdot \varepsilon +1\right)\cdot c}^{n}+{K_{d}}^{n}}$$ (5)

where the new parameter: q: the fraction of receptors that remained operable after the pretreatment with the irreversible antagonist. As compared to the Equation 4, the Equation 5 contains a small but important modification: its parameter ε is multiplied everywhere by q. The Equation 4 was only fitted to the “N” labelled data sets, while the Equation 5 was exclusively fitted to the “X” labelled ones. The two-model global fitting was performed first with shared ε, γ, n and q (among all data sets), and then ε and n were constrained to unity (providing the final results of the first fitting strategy).

For the second fitting strategy, only the data sets lacking FSCPX pretreatment were involved in the regression. The “N” labelled data sets were simultaneously fitted to the Equation 4 (one-model global fitting), initially with shared ε, γ and n, and then ε and n were constrained to unity.

For the third fitting strategy, the data sets generated with the same agonist were fitted at once. Similarly to the first fitting strategy, the Equation 4 was fitted to the “N” labelled data set, while the Equation 5 to the corresponding “X” labelled one. The regression was performed first with shared ε, γ, n and K_d_, and then ε and n were fixed at unity.

For the fourth fitting strategy, we returned to the arrangement used for the first one with the modification that all K_d_ values, provided by the third fitting strategy, were entered into the Equations 4, 5. This led to a six-model global fitting, where the modified Equations 4, 5 were fitted to the corresponding “N” and “X” labelled data sets, respectively (with a correspondence ensured by the appropriate K_d_ value). Importantly, all the six data sets were fitted at once with shared ε, γ, n and q (across all data sets), and then ε and n were constrained to unity here as well (see: Supplementary Appendix).

### 3.2 Regression with the operational model

The fitting of the operational model was performed using the equation as follows:

$$\frac{E}{E_{\max}}=\frac{{\left(c\cdot \tau \right)}^{n}}{{\left(c\cdot \tau \right)}^{n}+{\left(c+K_{d}\right)}^{n}}$$ (6)

where the new parameters: τ: the operational efficacy that characterizes the receptorial and postreceptorial components of signal handling as a single parameter; n: the operational slope factor (another Hill-type coefficient). The Equation 6 is a form of the classical equation of the operational model (Black and Leff, 1983) rearranged to handle the fractional effect instead of the effect (see: Supplementary Appendix). In the classical form, E_m_, K_A_ and n_op_ are used instead of E_max_, K_d_ and n, respectively, but with the same meaning.

### 3.3 E/c curve simulation

Using the Equation 4, six theoretical E/c curves were generated by combining two values for ε (0.17 and 1) with three values for γ (0.3, 1 and 136.8), while the constant parameters were n = 1 and logK_d_ = −5.93. Except for the arbitrary γ = 0.3 and γ = 1 (introduced to demonstrate the influence of γ on the shape and position of the E/c curves), parameter values were selected from the results provided by the third fitting strategy for data sets CPA N and CPA X directly (see below: Table 3B) or indirectly (ε = 0.17 came from q∙ε = 0.17∙1). Finally, for the sake of visualization, all simulated E/c curves were fitted to the Hill equation.

### 3.4 Data processing and presentation

Curve plotting and fitting were implemented with GraphPad Prism 10.4.2 for Windows (GraphPad Software Inc., La Jolla, CA, United States). Some calculations were made using Microsoft Excel for Microsoft 365 (Microsoft Co., Redmond, WA, United States).

The precision of regression was characterized by the width of the 95% confidence interval (CI) of the best-fit values. For computing 95% CIs, the “asymmetrical” option was always chosen. The precision of the curve fitting and the E/c curve data were characterized by the distance of the best-fit curve from the corresponding 95% confidence bands and the 95% prediction bands, respectively.

When setting the way in which the software checks how well the experimental data define the model, the option “Identify ambiguous fits” was chosen, because this way more estimates could be obtained.

The degree to which each variable parameter was intertwined with all the others was indicated by dependency, the value of which could range from 0 (independent parameter) to 1 (redundant parameter). According to the suggestion (Graphpad, 2025), dependency values greater than 0.9 and 0.99 were considered high and unacceptably high, respectively. If dependency was greater than 0.9999, the fitting software labelled the fit to be “ambiguous”.

The goodness of fit of the models was quantified by the coefficient of determination (R^2^) and its adjusted value. This latter is much lower than R^2^ if the model contains redundant parameters.

Regarding the E/c data set pairs constructed with the same agonist, the fit (correctness) of the SABRE and operational models was compared by means of the Akaike information criterion corrected for low sample size (AICc).

## 4 Results

### 4.1 Outcome of the first fitting strategy

Although 1) a relatively large amount of data to be reevaluated was present, 2) this database was quite diverse (in terms of the experimental design), and 3) four parameters were shared across the data sets (Figure 1), the estimates provided by curve fitting were marked as ambiguous for both the shared and unshared parameters. Accordingly, the CIs were reported to be very wide. To increase reliability even at the expense of accuracy, we constrained ε and n to unity/since NECA, CPA and CHA are widely thought to be full (or close to full) agonists, and because of our present observation that the fitted values of ε and n were close to unity (data not shown)/. Despite the constraints, the estimates and CIs remained ambiguous and very wide, respectively. Consistently, the dependency of all variable parameters was the maximum, i.e. 1 (Table 1).

**TABLE 1:** The main characteristics of the E/c relationship between the concentration of three synthetic A_1_ adenosine receptor full (or close to full) agonists (NECA, CPA, CHA) and the contractility of the isolated, paced guinea pig left atrium, determined with the SABRE model using two-model global fitting to all E/c data sets (with Equations 4, 5). The experiments were performed both in the absence (“N” labeling) and presence (“X” labeling) of a pretreatment with FSCPX, an irreversible A_1_ adenosine receptor antagonist (raw data obtained from Gesztelyi et al., 2013). During the final regression, parameters ε and n were constrained to unity, while parameters γ and q were shared among all data sets and the “X” labelled data sets, respectively. For each variable parameter, the best-fit value (top), confidence interval (middle) and dependency value (bottom) are presented. The statement “*very wide*” refers to the 95% confidence interval of the given best-fit value (provided by the fitting software).

	NECA N	CPA N	CHA N	NECA X	CPA X	CHA X
ε	= 1.00					
n	= 1.00					
γ	1.03					
	*very wide*					
	1.0000					
logK_d_	−7.70	−8.05	−7.33	−7.21	−7.32	−6.62
	*very wide*					
	1.0000					
K_d_	20.2 nM	8.9 nM	47.3 nM	61.7 nM	48.2 nM	240.8 nM
q	—	—	—	0.96		
				*very wide*		
				1.0000		
R^2^	0.9893	0.9838	0.9686	0.9355	0.9133	0.9737
Gl. R^2^	0.9612					
Adj. R^2^	0.9601					

E/c: concentration-effect; NECA: 5′-(*N*-ethylcarboxamido)adenosine; CPA: *N* ^*6*^-cyclopentyladenosine; CHA: *N* ^*6*^-cyclohexyladenosine; FSCPX: 8-cyclopentyl-*N* ^*3*^-[3-(4-(fluorosulfonyl)benzoyloxy)propyl]-*N* ^*1*^-propylxanthine; SABRE: Signal Amplification, Binding affinity, and Receptor-activation Efficacy (for an explanation of the SABRE parameters, see: Equations 4, 5); R^2^: the coefficient of determination; Gl. R^2^: the global coefficient of determination; Adj. R^2^: the adjusted coefficient of determination; nM: nmol/L.

Moreover, γ and K_d_, two important estimates, seem to be unrealistic. Our previous observation, i.e., FSCPX could not significantly decrease the maximum of the atrial direct negative inotropic response to full agonists of the A_1_ adenosine receptor (Gesztelyi et al., 2013; Viczjan et al., 2021), suggests a great receptor reserve for the investigated agonist-receptor-cell/tissue combination (viz. a strong signal amplification in this system) (Gesztelyi et al., 2013; Kiss et al., 2013; Zsuga et al., 2017). However, the γ estimate obtained from this regression was just above unity, indicating (in all likelihood wrongly) negligible postreceptorial signal amplification. Furthermore, considerably differing K_d_ estimates were obtained for the corresponding “N” and “X” labelled data sets, although K_d_ should have been the same for the same agonist-receptor-cell/tissue unit (in this regression arrangement, K_d_ could not be shared between the data sets generated with the same agonist). These observations also indicate that the first fitting strategy of the SABRE model failed (Table 1).

### 4.2 Outcome of the second fitting strategy

Assuming that the different K_d_ values obtained for the same agonists significantly contributed to the failure of the first fitting strategy, we repeated this kind of global fitting with the exclusion of the “X” labelled data sets, using only the Equation 4 (Figure 1). Neither the reliability nor the presumable reality of the estimates improved, indicating a complete failure of the simultaneous fitting to data sets constructed with different agonists (at least this way). Constraining ε and n to unity did not help either. Accordingly, the dependency of all variable parameters was 1, rendering this fit ambiguous as well (Table 2).

**TABLE 2:** The main characteristics of the E/c relationship between the concentration of three synthetic A_1_ adenosine receptor full (or close to full) agonists (NECA, CPA, CHA) and the contractility of the isolated, paced guinea pig left atrium, determined with the SABRE model using one-model global fitting (with the Equation 4) to the “N” labelled E/c data sets (i.e., constructed without a pretreatment with FSCPX, an irreversible A_1_ adenosine receptor antagonist; raw data obtained from Gesztelyi et al., 2013). During the final regression, parameters ε and n were constrained to unity, while parameter γ was shared among all data sets used here. For each variable parameter, the best-fit value (top), confidence interval (middle) and dependency value (bottom) are presented. The statement “*very wide*” refers to the 95% confidence interval of the given best-fit value (provided by the fitting software).

	NECA N	CPA N	CHA N
ε	= 1.00		
n	= 1.00		
γ	1.01		
	*very wide*		
	1.0000		
logK_d_	−7.70	−8.06	−7.34
	*very wide*		
	1.0000		
K_d_	19.8 nM	8.7 nM	46.3 nM
R^2^	0.9893	0.9838	0.9686
Gl. R^2^	0.9803		
Adj. R^2^	0.9799		

E/c: concentration-effect; NECA: 5′-(*N*-ethylcarboxamido)adenosine; CPA: *N* ^*6*^-cyclopentyladenosine; CHA: *N* ^*6*^-cyclohexyladenosine; FSCPX: 8-cyclopentyl-*N* ^*3*^-[3-(4-(fluorosulfonyl)benzoyloxy)propyl]-*N* ^*1*^-propylxanthine; SABRE: Signal Amplification, Binding affinity, and Receptor-activation Efficacy (for an explanation of the SABRE parameters, see: the Equation 4); R^2^: the coefficient of determination; Gl. R^2^: the global coefficient of determination; Adj. R^2^: the adjusted coefficient of determination; nM: nmol/L.

### 4.3 Outcome of the third fitting strategy

**FIGURE 2:**
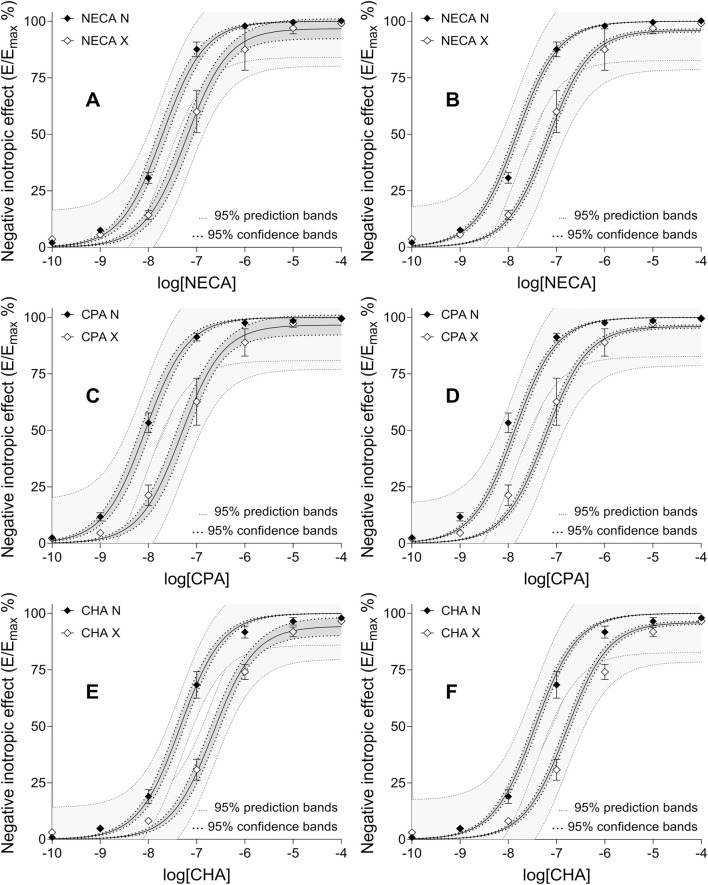
The E/c curves of three synthetic A_1_ adenosine receptor full (or close to full) agonists (NECA, CPA, CHA) generated in isolated, paced guinea pig left atria in the absence (filled symbols) and presence (open symbols) of a pretreatment with FSCPX, an irreversible A_1_ adenosine receptor antagonist (raw data obtained from Gesztelyi et al., 2013). The x-axis shows the common logarithm of the molar concentration of the given agonist, while the y-axis denotes the direct negative inotropic effect expressed as a percentage of the maximal effect achieved in this system (±SEM). The continuous lines show the best-fit curves of the SABRE model, fitted globally, using two arrangements: fitting simultaneously the E/c data set pairs constructed with the same agonist **(A, C, E)**, and fitting simultaneously all the six E/c data sets (but presented on three separate panels for clarity: **(B, D, F)**. For more details (sharing, constraints), see: Tables 3A–3C **(A, C, E)**, and Table 4 **(B, D, F)**. The thick dotted lines indicate the 95% confidence bands, while the thin dotted lines represent the 95% prediction bands. E/c: concentration-effect; SABRE: Signal Amplification, Binding affinity, and Receptor-activation Efficacy; NECA: 5′-(*N*-ethylcarboxamido)adenosine; CPA: *N* ^*6*^-cyclopentyladenosine; CHA: *N* ^*6*^-cyclohexyladenosine; FSCPX: 8-cyclopentyl-*N* ^*3*^-[3-(4-(fluorosulfonyl)benzoyloxy)propyl]-*N* ^*1*^-propylxanthine.

The breakthrough was brought about by the strategy to simultaneously fit only data sets related to the same agonist (Figure 1). When ε and n were fixed at unity, the third fitting strategy yielded substantially more reliable and realistic estimates than the two previous ones, for all three agonists (Figures 2A,C,E). The constraint of ε and n improved the outcome (in terms of both reliability and presumable reality), mostly for CHA (the weakest agonist here) and least for NECA (the strongest agonist here) (data not shown). Furthermore, the dependency of all variable parameters decreased below 0.99. Specifically, the dependency values of γ and logK_d_ were high but not unacceptable, while the dependency of q was moderate for all agonists. Thus, no fitting related to the third fitting strategy was ambiguous (Table 3).

**TABLE 3:** The main characteristics of the E/c relationship between the concentration of NECA (Table 3A), CPA (Table 3B) and CHA (Table 3C), three synthetic A_1_ adenosine receptor full (or close to full) agonists, and the contractility of the isolated, paced guinea pig left atrium. For the determination, the SABRE model (Equations 4, 5) and the operational model (the Equation 6) were globally fitted to the E/c data set pairs generated with the same agonist. The experiments were carried out with (“N” labeling) and without (“X” labeling) a pretreatment with FSCPX, an irreversible A_1_ adenosine receptor antagonist (raw data obtained from Gesztelyi et al., 2013). During the final regression using the SABRE model, parameters ε and n were constrained to unity, while parameters γ and logK_d_ were shared within each data set pair (generated with the same agonist). When fitting the operational model, the parameter n was constrained to unity, and logK_d_ was shared within each data set pair. For comparison, we present the earlier logK_d_ (plus K_d_) and q results (*in italics*) obtained from the same raw data by means of the operational model (fitted without any constraint) as well as Furchgott’s method (first published in: Gesztelyi et al., 2013). For each variable parameter, the best-fit value (top), confidence interval (middle) and dependency value (bottom) are presented. For technical reasons, confidence intervals published earlier (Gesztelyi et al., 2013) were computed with symmetrical approximation instead of the asymmetrical method used in the present study.

A	NECA N	NECA X	NECA N	NECA X	*NECA N*	*NECA X*	*NECA N*	*NECA X*
ε	= 1.00		op. m. (Y = E/Emax %)		*op. m. (Y = E)*		*Furchgott’s method*	
n	= 1.00		= 1.00					
γ	66.86							
	19.16 to ?							
	0.9885							
logK_d_	−5.88		−5.60		*−5.91*		*−5.83*	
	−6.40 to ?		−6.09 to ?		*−6.54 to −5.28*		*−5.94 to −5.73*	
	0.9870		0.9896					
K_d_	1.3 μM		2.1 μM		*1.2 μM*		*1.5 µM*	
q	—	0.31					*0.22*	
		0.21 to 0.45					*0.20 to 0.25*	
		0.5910						
R^2^	0.9893	0.9356	0.9892	0.9351				
Gl. R^2^	0.9655		0.9652					
Adj. R^2^	0.9646		0.9638					

B	CPA N	CPA X	CPA N	CPA X	*CPA N*	*CPA X*	*CPA N*	*CPA X*
ε	= 1.00		op. m. (Y = E/Emax %)		*op. m. (Y = E)*		*Furchgott’s method*	
n	= 1.00		= 1.00					
γ	136.80							
	41.47 to ?							
	0.9859							
logKd	−5.93		−5.87		*−5.30*		*−5.35*	
	−6.42 to ?		−6.31 to ?		*−7.37 to −3.23*		*−5.49 to −5.21*	
	0.9840		0.9807					
K_d_	1.2 μM		1.4 μM		*5.0 µM*		*4.5 µM*	
q	—	0.17					*0.11*	
		0.12 to 0.27					*0.10 to 0.12*	
		0.5943						
R^2^	0.9838	0.9134	0.9842	0.9134				
Gl. R^2^	0.9475		0.9477					
Adj. R^2^	0.9464		0.9466					

C	CHA N	CHA X	CHA N	CHA X	*CHA N*	*CHA X*	*CHA N*	*CHA X*
ε	= 1.00		op. m. (Y = E/Emax %)		*op. m. (Y = E)*		*Furchgott’s method*	
n	= 1.00							
γ	67.81							
	29.74 to 289.4							
	0.9770							
logK_d_	−5.51		−5.48		*−4.81*		*−4.66*	
	−5.84 to −4.88		−5.76 to −5.00		*−6.20 to −3.41*		*−4.79 to −4.52*	
	0.9717		0.9583					
K_d_	3.1 μM		3.3 μM		*15.6 µM*		*22.1 µM*	
q	—	0.20					*0.13*	
		0.14 to 0.27					*0.12 to 0.14*	
		0.6389						
R^2^	0.9686	0.9742	0.9701	0.9741				
Gl. R^2^	0.9715		0.9722					
Adj. R^2^	0.9708		0.9716					

E/c: concentration-effect; NECA: 5′-(*N*-ethylcarboxamido)adenosine; CPA: *N* ^*6*^-cyclopentyladenosine; CHA: *N* ^*6*^-cyclohexyladenosine; FSCPX: 8-cyclopentyl-*N* ^*3*^-[3-(4-(fluorosulfonyl)benzoyloxy)propyl]-*N* ^*1*^-propylxanthine; SABRE: Signal Amplification, Binding affinity, and Receptor-activation Efficacy (for an explanation of the SABRE parameters, see: Equations 4, 5); op. m. (Y = E/E_max_ %): the operational model fitted to data where y values were percentage effects (as in the case of the SABRE model); op. m. (Y = E): the operational model fitted earlier to data where y values were effects (Gesztelyi et al., 2013); R^2^: the coefficient of determination; Gl. R^2^: the global coefficient of determination; Adj. R^2^: the adjusted coefficient of determination; µM: µmol/L; ?: ambiguous value (according to the fitting software).

Values of γ were substantially greater than unity (they ranged from 66 to 137), indicating a strong postreceptorial signal amplification for all three agonists. (It should be noted that, as these γ values applied to the same process, i.e., postreceptorial signal handling, they might be expected to be the same.) K_d_ estimates were similar to those previously obtained with the operational model and Furchgott’s method (Gesztelyi et al., 2013), mostly for NECA and least for CHA (Table 3). Values of q varied over a relatively narrow range, from 0.17 to 0.31 (Table 3), which largely overlapped with the q range obtained earlier (0.11–0.22; Gesztelyi et al., 2013). (As the q values for all three agonists characterized the same event, i.e., FSCPX pretreatment, they should have been the same.)

### 4.4 Outcome of the fitting of the operational model

The results provided by fitting the operational model in the present investigation were similar to those obtained in our previous study (Gesztelyi et al., 2013). This was despite the fact that, in order to achieve a better match to the final regression of the SABRE model under the third fitting strategy, the n parameter of the operational model was fixed at unity (Table 3).

Based on AICc, the SABRE model seemed to be more correct than the operational model for NECA, but less for CPA and CHA. The values of relative probability of correctness were (SABRE vs. operational model) 58% vs. 42% for NECA, 46% vs. 54% for CPA and 22% vs. 78% for CHA, respectively. These (not very large) differences indicated the similar performance of the SABRE model and the operational model in terms of the information theory approach underlying AICc.

### 4.5 Experiences from the simulated E/c curves

**FIGURE 3:**
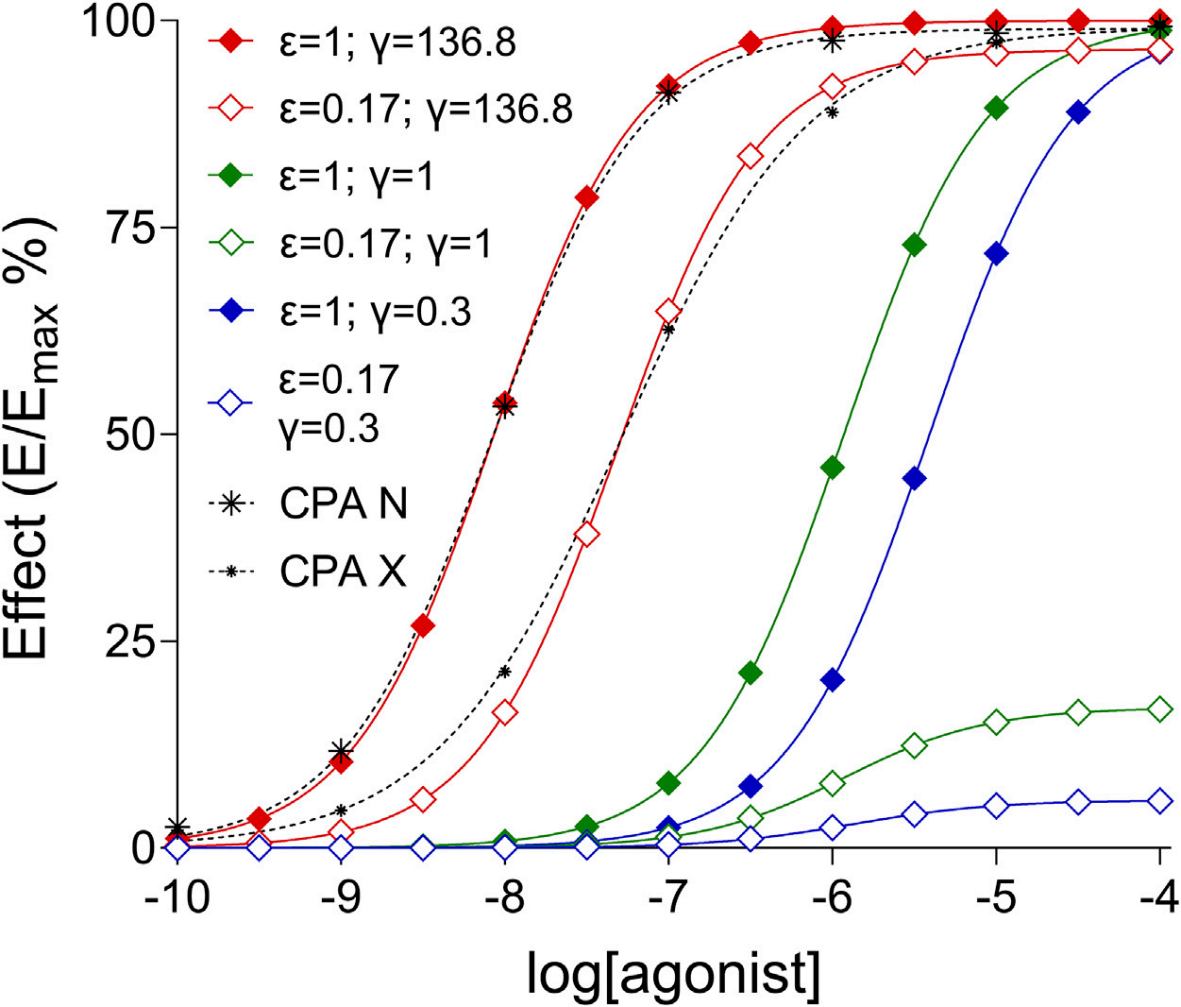
Six simulated and two *ex vivo* (CPA N, CPA X) E/c curves presented to demonstrate the influence of parameters ε and γ, defined within the SABRE model, on the shape of the E/c curves. The dashed black lines denote the fitted Hill equation to the *ex vivo* E/c curve data that display the direct negative inotropic effect of CPA, a synthetic A_1_ adenosine receptor full agonist, in isolated, paced guinea pig left atria, generated in the absence (CPA N) and presence (CPA X) of a pretreatment with FSCPX, an irreversible A_1_ adenosine receptor antagonist. The *ex vivo* effect values are normalized to the maximal effect achieved in this system (raw data were obtained from Gesztelyi et al., 2013). The continuous lines indicate the fitted Hill equation to the simulated E/c curve data of two hypothetical agonists, a partial (ε = 0.17; open symbols) and a full (ε = 1; filled symbols) one, acting in three systems with postreceptorial signal attenuation (γ = 0.3; blue curves), neutral postreceptorial signal handling (γ = 1; green curves) and postreceptorial signal amplification (γ = 136.8; red curves). The further SABRE parameters were n = 1 and logK_d_ = −5.93 (K_d_ ≈ 1.2 μM) for all simulated E/c curves. The values γ = 0.3 and γ = 1 were chosen arbitrarily, while the rest were selected from the results of the present study to reproduce the shape of the two *ex vivo* E/c curves shown here (see: Table 3B; Figure 2C). E/c: concentration-effect; SABRE: Signal Amplification, Binding affinity, and Receptor-activation Efficacy (for an explanation of the SABRE parameters, see: Equations 4, 5); CPA: *N* ^*6*^-cyclopentyladenosine; FSCPX: 8-cyclopentyl-*N* ^*3*^-[3-(4-(fluorosulfonyl)benzoyloxy)propyl]-*N* ^*1*^-propylxanthine.

The E/c curves, simulated with the Equation 4 using the final results provided by the third fitting strategy for data sets CPA N and CPA X (Table 3B), fitted well with the original *ex vivo* E/c curves of these data sets (Figure 3). As the SABRE model handles the phenomenon of partial agonism (ε < 1) analogously to the decrease in the number of operable receptors (i.e., with a multiplication of ε by a q < 1; cf. Equations 4, 5), the simulated E/c curve of the data set CPA X was identical to the E/c curve of the hypothetical partial agonist (ε = 0.17) in the system with strong postreceptorial signal amplification (γ = 136.8), which was produced for this simulation.

In the systems generated with arbitrary γ values modelling postreceptorial signal attenuation (γ = 0.3) and neutral postreceptorial signal handling (γ = 1), the distance between the E/c curves of the hypothetical full agonist (ε = 1) and the partial one (ε = 0.17) was considerably larger than that in the system with strong postreceptorial signal amplification (γ = 136.8) simulating our original *ex vivo* biological system. It was particularly spectacular that the strong postreceptorial signal amplification (γ = 136.8) allowed the E/c curve of a weak partial agonist (ε = 0.17) to reach almost the same maximum as the E/c curve of a full agonist (ε = 1) (Figure 3). This finding is in accordance with our previous conclusion that the inability of FSCPX to reduce the maximal inotropic response to A_1_ adenosine receptor full agonists in the guinea pig atrium indicates great receptor reserve for the direct negative inotropic effect of the investigated full agonists mediated by the guinea pig atrial A_1_ adenosine receptor (Gesztelyi et al., 2013; Kiss et al., 2013; Zsuga et al., 2017).

### 4.6 Outcome of the fourth fitting strategy

To take full advantage of the SABRE model, we repeated the simultaneous fitting to all data sets (as done during the first fitting strategy, see: Figure 1), but fixing K_d_ at the values yielded from the third fitting strategy (Figures 2B,D,F). Constraining ε and n to unity changed the values of the remaining variable parameters only to a slight extent and only moderately improved the already good reliability of the estimates (data not shown). The values of γ and q obtained with the fourth fitting strategy were within the ranges denoted by the minimal and maximal values provided by the third fitting strategy for the three agonists. In addition, the q value of the fourth fitting strategy was the upper limit of the q range obtained in our previous study (Gesztelyi et al., 2013). The dependency of γ and q (these two values coincided) was smaller than the dependency values provided by the third fitting strategy, indicating a high level of reliability for the results of the fourth fitting strategy (Table 4). In addition, the adjusted R^2^, which was otherwise only slightly smaller than *R* ^2^ throughout the entire study, was particularly close to R^2^ when applying the fourth fitting strategy (Tables 1–4).

**TABLE 4:** The main characteristics of the E/c relationship between the concentration of three synthetic A_1_ adenosine receptor full (or close to full) agonists (NECA, CPA, CHA) and the contractility of the isolated, paced guinea pig left atrium, determined with the SABRE model using six-model global fitting to all E/c data sets (with Equations 4, 5, after constraining their logK_d_ parameters to the relevant values shown in Table 3; see: Supplementary Appendix). The experiments were performed in the absence (“N” labeling) and presence (“X” labeling) of a pretreatment with FSCPX, an irreversible A_1_ adenosine receptor antagonist (raw data obtained from Gesztelyi et al., 2013). During the final regression, parameters ε and n were fixed at unity, while parameters γ and q were shared among all data sets and the “X” labelled data sets, respectively. For each variable parameter, the best-fit value (top), confidence interval (middle) and dependency value (bottom) are presented.

	NECA N	CPA N	CHA N	NECA X	CPA X	CHA X
ε	= 1.00					
n	= 1.00					
γ	86.84					
	75.05 to 100.40					
	0.5047					
q	—	—	—	0.22		
				0.18 to 0.27		
				0.5047		
R^2^	0.9851	0.9700	0.9653	0.9349	0.9103	0.9655
Gl. R^2^	0.9558					
Adj. R^2^	0.9556					

E/c: concentration-effect; NECA: 5′-(*N*-ethylcarboxamido)adenosine; CPA: *N* ^*6*^-cyclopentyladenosine; CHA: *N* ^*6*^-cyclohexyladenosine; FSCPX: 8-cyclopentyl-*N* ^*3*^-[3-(4-(fluorosulfonyl)benzoyloxy)propyl]-*N* ^*1*^-propylxanthine; SABRE: Signal Amplification, Binding affinity, and Receptor-activation Efficacy (for an explanation of the SABRE parameters, see: Equations 4, 5); R^2^: the coefficient of determination; Gl. R^2^: the global coefficient of determination; Adj. R^2^: the adjusted coefficient of determination.

## 5 Discussion

The first step in regression is to choose a proper model (equation), hopefully the best one. If none of the available models are really good, it is worth trying to develop a new model that is better than the older ones in at least one aspect. Importantly, any model chosen for regression implies some interpretation of the data to be evaluated. In turn, a model can be optimized regarding two main aspects: to achieve the best fit (i.e., towards an empirical model, for which interpretation is less important), and to extract the most meaningful pieces of information about the underlying mechanisms (sc. Towards a mechanistic model, in which interpretation is central) (Motulsky and Christopoulos, 2004). The recently developed SABRE model, the newest general and quantitative model of receptor function (Buchwald, 2017; Buchwald, 2019; Buchwald, 2020; Buchwald, 2022; Buchwald, 2023; Buchwald, 2025), corresponds visibly to the latter aspect.

A unique feature of the SABRE model is to distinguish between receptor activation (accounted for by parameter ε) and activation of postreceptorial signaling (characterized by parameter γ). Although the two phenomena (expressed by ε and γ) are logically (and didactically) well separable (cf. Equations 2, 3), their integration into one model is a challenge. In the SABRE model, this integration has been solved in a relatively complex way (see: the Equation 4), reflecting the fact that these two phenomena are closely related. The goal of the present study was to test the capabilities of a novel method (Buchwald, 2022) based on this new model (Buchwald, 2017; Buchwald, 2019), which enables the determination of K_d_ (the apparent equilibrium dissociation constant of the agonist-receptor complex) and q (the fraction of the operable receptors after a partial irreversible receptor inactivation) from purely functional data.

For the present study, we used data from one of our previous investigations that contained *ex vivo* E/c curves generated at different receptor levels (Gesztelyi et al., 2013). These data challenged the classic functional assays to determine K_d_, because the maximal response of the receptor-depleted tissue was practically the same as that of the naïve tissue. The preservation of the maximal response was explained by assuming a great receptor reserve (signal amplification) in terms of the investigated agonists (NECA, CPA, CHA), receptor (A_1_ adenosine receptor), tissue (guinea pig left atrial myocardium) and effect measured (direct negative inotropy). We were and are aware that the preserved maximal response after partial irreversible receptor inactivation might negatively affect the results of both the previous study (Gesztelyi et al., 2013) and the present investigation. For a reliable evaluation, the maximal effect after partial irreversible receptor inactivation is thought to have to be significantly smaller than the original maximal effect (Motulsky and Christopoulos, 2004).

In our previous study (Gesztelyi et al., 2013), two accepted functional assays, the operational model of agonism (Black and Leff, 1983) and Furchgott’s method (Furchgott, 1966; Furchgott and Bursztyn, 1967) were implemented. The K_d_ values determined for the same agonist with these methods were similar (Table 3). Furthermore, the three q values, which were determined with Furchgott’s method using the three agonists, were also close to one another (an observation that was otherwise expected) (Table 3). These findings might suggest that the results of our previous study are reliable. Nevertheless, it should serve as a warning that the K_d_ values obtained are considerably higher than those reported by others using different methods, including ligand binding assays and Furchgott’s method (Gesztelyi et al., 2013).

During the reevaluation of our challenging data by means of the SABRE model, similar K_d_ values to those supplied by the two previously used older methods were finally obtained. The q values provided by the SABRE model were also similar to the earlier ones obtained with Furchgott’s method. These results allow for two possible interpretations. On the one hand, it may be assumed that all estimates obtained with these three methods are reliable. On the other hand, the challenging data may have biased the determination made with the SABRE model in the same direction as they did in the case of the two older methods. If this second assumption is true, it may be concluded that the SABRE model, similarly to the rival methods, is demanding of data quality. This conclusion is supported by the observation that less prudent fitting ways have led to unreliable (and unrealistic) results (Figure 1; Tables 2, 3). Consistent with this, it is of paramount importance to find the most suitable fitting strategy (or strategies) for the SABRE model (Figures 1, 2; Tables 1, 4).

It is worth noting that the third fitting strategy provided different γ values for the different agonists (Table 3). Since γ characterizes the postreceptorial signal handling, the same γ value might be expected for the direct negative inotropy mediated by the guinea pig atrial A_1_ adenosine receptor, irrespective of the agonist used. However, it has been established that NECA, CPA and CHA activate the same signaling pathways of the A_1_ adenosine receptor, but to somewhat different extents (Verzijl and Ijzerman, 2011). This fact might influence the γ values obtained with the third fitting strategy (Table 3). Of course, these differences disappeared with the fourth fitting strategy, which assigned a single γ value to the three agonists *a priori* (Table 4).

It should also be noted that, owing to the flexibility of the SABRE model, it is possible to implement regression using several arrangements (Figure 1) including ones that incorporate the results of previous fittings (even using the same database). Utilizing the K_d_ values obtained with the third fitting strategy (which proved to be the best for this task), the fourth fitting strategy (a modified version of the first one) offered the most reliable estimates for the other parameters (Figures 2B,D,F; Table 4). This is indicated by the following findings: the estimates obtained with the fourth fitting strategy had 1) the narrowest CIs and 2) the smallest dependency values (cf. Tables 1–4); furthermore 3) the difference between R^2^ and its adjusted value was the smallest for the fourth fitting strategy (cf. Tables 1–4); and 4) the best-fit curves were closest to their confidence (but not prediction) bands in the case of the fourth fitting strategy (cf. Figures 2A–F).

A possible reason for difficulties in using the SABRE model is its innovation, i.e., distinction between ε and γ, which increases the number of parameters. Moreover, ε and γ refer to two consecutive steps (the activation of the receptor and the activation of the postreceptorial signaling) of the same process (signal handling), which may increase the potential intertwining between these two parameters during regression. Each of these can be a source of uncertainty. It is widely recognized that increasing the complexity of a model (used for regression) tends to increase the correlation (intertwining) among the parameters, which reduces the accuracy and reliability of the estimates (Motulsky and Christopoulos, 2004; Graphpad, 2025). Regarding ε and γ, it seems to be an obvious solution to constrain one of them to a reasonable value (Buchwald, 2017; Buchwald, 2019; Buchwald, 2020; Buchwald, 2022; Buchwald, 2023). However, this practice has not always proved sufficient (Tables 2, 3).

In contrast, the complexity of the SABRE model, which can be a disadvantage when fitting experimental data, becomes an advantage when simulating ligand-receptor-cell/tissue interactions. The unique parametrization of the SABRE model provides a wider range of possibilities than ever before to investigate different combinations of ligand and/or biological system properties *in silico* in order to test hypotheses or to explain experimental observations (Buchwald, 2017; Buchwald, 2019; Buchwald, 2020; Buchwald, 2022; Buchwald, 2023; Buchwald, 2025) (Figure 3).

There are several approaches to compare different mathematical models regarding their utility in data evaluation. However, most of them are only valid to compare related (nested) models or require a much larger database than ours. Therefore, we had to settle for AICc, which is suitable for comparing non-related models even with limited data. This can be considered a limitation of the present study.

In conclusion, the present study has demonstrated that the most recent method to determine K_d_ values for agonist-receptor pairs from purely functional data (Buchwald, 2022), which is based on the SABRE model, the newest general and quantitative receptor function model (Buchwald, 2017; Buchwald, 2019), is at least as useful as two widely accepted, older methods thought to have similar capabilities, namely, the operational model of agonism (Black and Leff, 1983) and Furchgott’s method (Furchgott, 1966; Furchgott and Bursztyn, 1967). Nevertheless, the method based on the SABRE model requires a large amount of high-quality and, regarding the experimental setup, diverse data to provide reliable results, when evaluating purely functional data. In addition, it is important to find the best way to fit the equation(s) of the SABRE model to the particular sort of data. However, the SABRE model, owing to its unique feature to distinguish between the activation of the receptor and the activation of the postreceptorial signaling, appears to be superior to the earlier quantitative receptor function models to simulate E/c curves and thereby to clarify, explain or simply visualize theoretical issues.

## Data Availability

The raw data supporting the conclusions of this article will be made available by the authors, without undue reservation.
