# Supplementary material for: The flexibility of SABRE, a new quantitative receptor function model, when fitting challenging concentration-effect data
Source: Front Pharmacol. 2025 Jun 12;16:1591761. doi: 10.3389/fphar.2025.1591761 (PMC12198161; doi:10.3389/fphar.2025.1591761)
Supplement: Supplementary file 1 [file Supplementaryfile1.docx]

# Supplementary appendix

As Hill equation, the software's built-in equation “log(agonist) *vs.* response - Variable slope (four parameters)”, with a “bottom” parameter constrained to zero, was used. The other equations are presented as they were fitted by the curve fitting software. Their independent variable was logc (indicated here as X), and the dependent variable was E/E_max_ % (indicated here as Y or the name of the given equation, written with capital letters). The number 100 (instead of 1) in all equations below transforms them to handle percentage effect (instead of fractional effect).

For the first fitting strategy, the following multiline model was applied (two-model global fitting):

**SABRE=100*(ε*γ*10^(n*X))/((ε*γ-ε+1)*10^(n*X)+10^(n*logKd))**

**SABREq=100*(q*ε*γ*10^(n*X))/((q*ε*γ-q*ε+1)*10^(n*X)+10^(n*logKd))**

**<A:C>Y=SABRE**

**<D:F>Y=SABREq**

For the second fitting strategy, the following equation was used (one-model global fitting):

**Y=100*(ε*γ*10^(n*X))/((ε*γ-ε+1)*10^(n*X)+10^(n*logKd))**

For the third fitting strategy, the following multiline model was used (two-model global fitting):

**SABRE=100*(ε*γ*10^(n*X))/((ε*γ-ε+1)*10^(n*X)+10^(n*logKd))**

**SABREq=100*(q*ε*γ*10^(n*X))/((q*ε*γ-q*ε+1)*10^(n*X)+10^(n*logKd))**

**<A>Y=SABRE** or **<B>Y=SABRE** or **<C>Y=SABRE**, for NECA or CPA or CHA data sets, respectively

**<D>Y=SABREq** or **<E>Y=SABREq** or **<F>Y=SABREq**, for NECA or CPA or CHA data sets, respectively

For the fourth fitting strategy, the following multiline model was used (six-model global fitting):

**NECA=100*(ε*γ*10^(n*X))/((ε*γ-ε+1)*10^(n*X)+10^(n*(-5.882)))**

**NECAq=100*(q*ε*γ*10^(n*X))/((q*ε*γ-q*ε+1)*10^(n*X)+10^(n*(-5.882)))**

**CPA=100*(ε*γ*10^(n*X))/((ε*γ-ε+1)*10^(n*X)+10^(n*(-5.927)))**

**CPAq=100*(q*ε*γ*10^(n*X))/((q*ε*γ-q*ε+1)*10^(n*X)+10^(n*(-5.927)))**

**CHA=100*(ε*γ*10^(n*X))/((ε*γ-ε+1)*10^(n*X)+10^(n*(-5.506)))**

**CHAq=100*(q*ε*γ*10^(n*X))/((q*ε*γ-q*ε+1)*10^(n*X)+10^(n*(-5.506)))**

**<A>Y=NECA**

**<D>Y=NECAq**

**<B>Y=CPA**

**<E>Y=CPAq**

**<C>Y=CHA**

**<F>Y=CHAq**

For fitting the operational model, the following rearranged model was used (one-model global fitting to the E/c data set pairs generated with the same agonist):

**Y=100/(1+((10^logKd+10^X)/10^(logtau+X))^n)**
